# The Role of Estrogen in the Treatment of Men with Schizophrenia

**DOI:** 10.5812/ijem.6615

**Published:** 2013-07-01

**Authors:** Jayashri Kulkarni, Emmy Gavrilidis, Roisin Worsley, Tamsyn Van Rheenen, Emily Hayes

**Affiliations:** 1 The Monash Alfred Psychiatry Research Centre, The Alfred and Monash University School of Psychology, Psychiatry and Psychological Medicine, Melbourne, Australia

**Keywords:** Estrogen, Schizophrenia, Psychosis, Neuroprotection, Men, Selective Estrogen Receptor Modulators

## Abstract

Schizophrenia is a debilitating and pervasive mental illness with devastating effects on many aspects of psychological, cognitive and social wellbeing. Epidemiological and life-cycle data point to significant differences in the incidence and course of schizophrenia between men and women, suggesting that estrogen plays a “protective” role . Adjunctive estrogen therapy has been shown to be effective in enhancing the treatment of schizophrenia in women. In men, consideration of estrogen therapy has been impacted by concerns of feminisation, however, clinical trials using estrogen to treat prostate cancer, bone density loss and even aggression in men with dementia or traumatic brain injury, show estrogen to be a safe and effective therapy. Findings do, however, suggest that further exploration of a therapeutic role for adjunctive estradiol treatment in men with schizophrenia is warranted. The development of the new estrogen compounds - Selective Estrogen Receptor Modulators (SERMs) which do not cause feminisation - opens up the possibility of using a different type of estrogen for a longer period of time at higher doses. Estrogen could therefore prove to be an important component in the treatment of psychotic symptoms in men with schizophrenia. This review explains the scientific rationale behind the estrogen hypothesis and how it can be clinically utilised to address concerns unique to the care of men with schizophrenia.

## 1. Background

Schizophrenia is a psychotic illness characterised by a constellation of positive and negative symptoms, resulting in significant social and/or occupational impairment (1). In Australia, it is estimated that approximately 5 in 1000 adults suffer from a psychotic illness such as schizophrenia, with the prevalence being slightly higher in males than females (2). Although the prevalence is low in comparison to other psychiatric disorders such as depression and anxiety, the burden of disease for the individual and family plus the financial cost to the community are incredibly high and not to be underestimated (3). Additionally, a significant number of patients with schizophrenia do not recover with standard forms of pharmacologic treatment, or develop serious adverse effects that further debilitate their already poor quality of life. Therefore, safe and effective augmentation strategies need to be urgently identified and investigated. Adjunctive estrogen therapy could be one such strategy.

While the possible link between sex hormones such as estrogen, and schizophrenia was considered as early as the late 19th century by luminaries such as Kraepelin and Krafft-Ebing (4, 5) it is only in recent decades that extensive preclinical research has begun to substantiate this link. Animal and human studies have demonstrated the profound effects that sex hormones can exert in the central nervous system, including the neurotransmitter systems thought to underlie the pathogenesis of schizophrenia. Such research has led multiple authors (6-9) to propose that estrogen may therefore protect against schizophrenia, and in fact, clinical trials to date on the use of adjunctive estradiol therapy in women with schizophrenia have been promising (10, 11). This paper aims to explore whether adjunctive estrogen therapy could be an equally viable treatment option in men.

## 2. Gender Differences in Schizophrenia Symptoms and Course of Illness

Differences in the time course of schizophrenia between men and women underlies the ‘estrogen hypothesis’ of schizophrenia (12). Although lifetime prevalence rates of schizophrenia in males and females are relatively similar (13, 14), recent meta-analyses have demonstrated that the incidence of schizophrenia in men is actually approximately 1.5 times higher than in women (15, 16). More importantly, the average age of onset in men is considerably younger than for women: for males the mean age of onset is 26.5 years, whereas in females, it is 30.6 years (6). It is hypothesised that in women, estrogen may act as a psychoprotective agent both during the early stages of brain development and following pubertal increases in sex steroids. Estrogen action thus accounting for the lower incidence and later onset of schizophrenia in pre-menopausal females in comparison to their male counterparts (17).

First-episode late onset schizophrenia is more common in women than men (18), often occurring in women during the perimenopause, a time of fluctuating and eventually declining estrogen levels. This is again consistent with the notion that estrogen may offer women some protection against psychotic illness – a protection which is lost with the diminishing estrogen levels seen at the menopause. In some women, symptoms are also worse during the low-estrogen early-follicular phase of the menstrual cycle (19, 20). Marked differences in course, severity and expression of illness also exist between the sexes. Being diagnosed at a younger age, men often have not yet attained the same degree of social development as women at onset of illness (21). This combined with the observation that men tend to display significantly higher levels of adverse illness behaviours and substance abuse (22), contributes to the poorer social outcomes of men with schizophrenia. Furthermore, many authors report differences in symptomatology for males and females. For example, early studies found that men were more likely to experience negative and depressive symptoms than women, who were instead more likely to display symptoms of paranoia (23, 24). In a population study conducted by Dopfner and colleagues, conduct disorders, aggressive behaviours, antisocial personality traits and drug abuse were more often present in men (25). More recent, larger studies have demonstrated that in general, men experience a significantly more severe course of illness than women overall, exhibiting higher levels of psychopathology and disability, and lower levels of functioning, treatment adherence, response to antipsychotic medications and insight (22, 26). Limited insight in particular may lead to poorer outcomes for men as it can delay and impair treatment (27).

## 3. Psychoprotective Properties of Estrogens 

Estrogens are neurosteroids with widespread genomic and non-genomic effects (28, 29). In men, estrogen in the form of estrone (E1) and estradiol (E2) is derived from the conversion of testosterone by the enzyme aromatase. Estradiol has a stronger affinity for estrogen receptors and is the isoform used in clinical practice.

The effects of estrogens on cognition and mood have been well established (e.g. (30-34)). Estrogen receptors are densely populated in areas of the human brain that control cognitive and emotional function. These include subcortical regions such as the hippocampus (memory) and amygdala (emotion) as well as a range of cortical areas involved in higher order functioning (35-38).

Estrogens modulate the neurotransmitters responsible for cognitive and emotional processes (39). For example, estrogens reduce dopamine receptor sensitivity and increase D2 receptor density in the striatum of ovariectomised rats, while also significantly enhancing serotonergic neurotransmission (40-43). Its effects on mental state are thought to occur via an influence on the availability of these mood-relevant neurotransmitters in the synapse (44-47). In particular, estrogens appear to have a protective effect on neurodegenerative disorders characterised by core cognitive dysfunctions including Parkinson’s disease and Alzheimer’s disease. The cardinal symptoms of Parkinson’s disease are caused by dopaminergic cell death, yet estradiol appears to have an anti-apoptotic effect on dopaminergic neurons (48). Thus, increased estrogen availability in females may delay the onset of Parkinson’s by increasing levels of striatal dopamine at symptom onset (49).

In Alzheimer’s disease, neuropsychological function has also been linked with reduced dopamine activity (50). Estrogen use in both Parkinson’s and Alzheimer’s populations have however been associated with lower symptom severity, better cognitive functioning and greater independence in activity (50-54). Moreover, women with a history of estrogen use post menopause demonstrate reduced Alzheimer’s risk and later disease onset (55, 56). Substantial reductions in estrogen levels have also been found in the postmortem brains of females with Alzheimers compared with age and gender-matched controls (57, 58).

To date, research into the effects of estrogens on cognition and mood has predominantly focused on women. Increased estrogen levels are associated with reduced negative affect and heightened wellbeing, as well as enhanced cognitive function including improved fluency, processing speed and better verbal and motor skills in healthy women (59).

The protective effect of estrogen in schizophrenia is well researched and is hypothesised to be the result of its neuroleptic-like effect on the dopamine system (19, 60-63). Dopamine overproduction is a pathophysiological feature of the illness and estrogen appears to blunt this effect by reducing dopamine receptor sensitivity and increasing the threshold for vulnerability (64, 65). Interestingly, neurocognitive and mood impairments are features of schizophrenia and other severe mental illnesses characterised by abnormalities in dopaminergic and serotonergic activity (66-75). Increased estrogen appears to have a positive effect on these symptoms and abilities. For example, in schizophrenic women during periods when estrogen levels are high, cognitive and positive symptoms have been found to decrease (76). Higher levels of estrogen have been associated with better executive function (77) and verbal and spatial memory (78) as well as decreases in psychotic symptoms (11, 62). Estrogen augmentation has also been found to be effective in improving mood symptoms in women suffering various depressive disorders (31, 32).

In men, the estrogen literature is less comprehensive and its effect on cognition and mood is not particularly well understood. Some research has demonstrated a relationship between higher estrogen levels and better cognitive performance in males (79-83). However evidence remains mixed, with other studies failing to find associations with disorders of cognition (84) and others still, reporting decreases in cognitive ability when estrogen levels are high (85-87).

Poor cognition and depressive symptoms have been found in men with low testosterone levels (88-93). Testosterone supplementation on the other hand, has been associated with improvements in these functions (94, 95) and is postulated to be neuro-protective in men with disorders characterised by cognitive dysfunction (96, 97).

Testosterone deprivation, which results in low estrogen levels, has been related to psychosis (98), deficits in spatial ability and memory (99) and increases in anxious and depressive symptoms (100). Improvements in spatial and verbal memory as well as executive integration have been observed in men receiving testosterone supplementation where estrogen levels in the testosterone treated groups were substantially increased (95, 101). Collectively these findings imply that the effect of testosterone on cognition and mood may well be due to estrogen.

In animal studies estrogen has been found to increase the density of serotonin binding sites in higher brain regions including the frontal cortex and has been implicated in the acquisition and improvement of spatial learning and memory (102-104). In human males, verbal memory improvements in those administered testosterone are negated by the coadministration with an aromatase inhibitor that prevents the conversion of testosterone to estrogen (105). These findings fit well with reports of better verbal memory performance in male to female transsexuals undergoing estrogen therapy when compared to those awaiting it, and better visual memory performance in men with heightened circulating estrogen levels compared to menopausal women (106, 107). Higher estrogen levels have also been associated with lower depressive symptoms in elderly patients of both sexes (108).

Estrogen supplementation in men is relatively rare, although it has been used in the treatment of prostate cancer and there are growing reports of its effectiveness in improving testosterone deficiency related verbal memory loss in men, reducing cardiovascular toxicity and male vasomotor symptoms, increasing bone density and improving quality of life (93, 109-111). In schizophrenia, estrogen levels in men tend to be low and estrogen augmentation may prove effective and have similar protective benefits for men as it does for women with the disorder.

Recently, our group demonstrated the effectiveness of oral estrogen therapy in the treatment of psychopathological symptoms in males with schizophrenia (112). These findings corroborate an earlier pilot study of estrogen therapy in men with schizophrenia, as well as reports of a reduction in depressive, aggressive, anxiety and psychotic symptoms in elderly men and women with dementia when treated with estrogen (113-115). These findings highlight the therapeutic efficacy that estrogen may entail for mental illnesses characterised by cognitive and mood dysfunction. However, further research is needed to explicitly examine its role in the treatment of severe mental illnesses in men.

## 4. Concerns Regarding the Use of Estradiol in Men

Use of estradiol in men is limited by side effects, particularly feminisation. The dose used for the development of female secondary sexual characteristics in male to female transsexuals is 2-4mg of oral estradiol valerate (or transdermal E2) (116) combined with potent antiandrogen therapy. In contrast, studies assessing the use of estradiol in psyhiatric illness use in the order of 1mg estradiol and no antiandrogen therapy.

In psychiatric studies using lower doses of estrogen over short periods of time side effects leading to withdrawal from the trials have included nausea, headaches and gynecomastia, (117) symptoms similar to those that cause cessation of hormone therapy in women.

The risk of venous thrombosis is another concern of long term use of estrogen therapy. Oral estrogen therapy is known to increase the risk of deep vein thrombosis in pre and post menopausal women, via increased production of clotting factors by the liver, due to first pass metabolism. In fact, estrogen therapy has been used to treat bleeding problems in both men and women with renal failure and other disorders (118). The Women’s Health Initiative found that combined estrogen and progestin therapy double the risk of venous thromboemolism (VTE), whilst oral estrogen only therapy increased the risk by 32% in postmenopausal women (119). Transdermal estradiol however, does not appear to increase the risk of venous thromboembolism in postmenopausal women (120). The oral contraceptive pill increases the risk of VTE by more than two fold in women between 15-44 years old, (121) though the level of risk varies with the progestin used.

Whether estrogen therapy in men could increase rates of hormone dependent cancer is another concern. In a Dutch study, 966 male to female transsexual men from one gender dysphoria clinic were followed up after a median of 18.5 years (116). While overall mortality was increased compared with the general population, through increased deaths related to suicide, drugs, AIDS and cardiovascular disease, there was no increase in the overall rate of cancer mortality. No cases of breast cancer were observed (116). This is reassuring although the results are not generalisable to other populations and the group size prevents the detection of small increases in risk (as in the Womens Health Study).

Estrogen plays a key role in male fertility. Circulating estrogens as well as locally produced estrogen (from aromatization of testosterone in local tissues) suppresses gonadotrophin releasing hormone (GnRH) at the level of the hypothalamus and pituitary (119) Administration of aromatase inhibitors to adult men (with a resultant decrease in estradiol and increase in testosterone) results in raised LH and FSH. Chronic low dose estrogen therapy for schizophrenia could therefore result in hypogonadotrophic hypogonadism, with potentially lower serum testosterone levels.

Whether estrogens play a role in spermatogenesis is not understood. Concerns have been raised that the decline in human fertility in developed societies in recent decades may be attributable to exposure to xeno-estrogens (120).

Selective estrogen receptor modulators (SERMs) are compounds that can have both estrogen receptor agonist and antagonist actions. The hope is that these agents will be able to provide men with the benefits of estrogen in the brain without the peripheral feminizing effects.

In large studies of the SERM raloxifene in women, an increased risk of venous thromboembolism and fatal stroke has been found (122-124). These studies used doses of 60mg orally, half the dose required for a therapeutic effect in schizophrenia.

The risks of estrogen and SERM therapy in men is largely unknown. In our studies we are particularly vigilant for signs of feminisation, and venous thromboembolism. In the longer term, we will need to examine the potential effects on fertility and stroke risk. Ischaemic stroke has been associated with estrogen therapies in women, including the combined oral contraceptive pill,(125) tibolone (a hormone like therapy), (126) post menopausal hormone therapy (127) and raloxifene (128). This is of particular concern in the treatment of schizophrenia as the rates of stroke risk factors such as smoking are considerably high in men and women with schizophrenia (129).

## 5. Selective Estrogen Receptor Modulators

SERMs are a class of compounds that includes the commonly used drugs raloxifene and tamoxifen and newer agents such as bazedoxifene and lasofoxifene. Raloxifene hydrochloride is a SERM, that acts as a tissue specific estrogen receptor agonist or antagonist (130). It is a benzothiophene derivative, and is distinguished from SERM compounds of the triphenylethylene class, including tamoxifen and toremifene, by its lack of endometrial stimulation (131). Like other drugs of its class, it is also antiproliferative in the breast (122) and prevents bone loss due to its agonist estrogenic action in bone (132). Most importantly, raloxifene has agonist actions in the brain similar to those of natural estrogen, offering positive estrogenic effects on neurotransmitter and neuronal systems (133). It appears to influence multiple neurotransmitter pathways being able to cross the blood-brain barrier in sufficient quantities to have a measurable effect in humans. Some of the neurotransmitter systems that may be influenced by raloxifene include serotonergic and dopaminergic systems in the frontal cortex, striatum and basal ganglia (134), brain areas commonly altered in people with schizophrenia (135). The SERMs appear to have an estradiol-like effect on brain neurochemistry, but without the adverse impact on breast and uterine tissue. We are currently conducting a large study using the SERM raloxifene as an adjunctive treatment in women with schizophrenia and our early, promising results have been published (136). SERMs have more targeted central nervous system actions, with minimal actions on estrogen receptors in breast or other sex organ tissue (137). Clinical use of SERMs may therefore enable improved targeting of activity in key brain regions associated with positive and negative psychotic symptoms without the risk of feminizing side effects.

The effects of raloxifene on brain function are still under investigation, and likely to be complex. Animal studies have suggested that raloxifene demonstrates estrogen-like beneficial effects on cholinergic transmission within the hippocampus (138), lending support for raloxifene to potentially improve aspects of cognitive function in particular. Of the few studies to explore the cognitive enhancing effects of raloxifene, the results of the MORE trial (5386 postmenopausal women with osteoporosis) found that compared to placebo, raloxifene at a dose of 120 mg/day, but not 60 mg/day, resulted in a reduced risk of developing mild cognitive impairment in post menopausal women (139). Data from smaller randomised trials have demonstrated slight improvements in verbal memory performance after one month of raloxifene (120 mg/day) treatment in postmenopausal women with osteoporosis (140), and after 12 weeks of raloxifene (120 mg/day) treatment in women with Alzheimer’s disease (141).

A study on the effect of raloxifene on cognition in healthy, postmenopausal women found a slight increase in verbal memory performance after one month of high dose treatment, while no other differences were found after 12 months of treatment (140). A recent cognition and estrogen study showed a positive correlation between improved cognition and higher estrogen levels (142). Yaffe et al. (2005) trialled two doses of raloxifene (60 mg oral per day and 120 mg oral per day) vs. placebo in postmenopausal women with normal cognition, mild cognitive impairment and Alzheimer’s disease (139). The women receiving 120 mg raloxifene per day had better cognitive outcomes compared to the 60 mg raloxifene per day group or the placebo group.

## 6. Conclusions

Schizophrenia is a serious and debilitating disease with significant social and economic costs to the individual, their family and society. New treatment approaches are needed to improve patients’ quality of life and functioning above the current outcomes seen with existing treatments. Innovative adjunctive hormone therapies, such as estradiol and SERMs, may offer new opportunities to achieve improved outcomes in a broad population of individuals with schizophrenia. The development of non-feminising estrogens, SERMs, opens up a potentially new and safe treatment strategy for men suffering under the considerable burden of schizophrenia. Further research is needed to assess the efficacy of short term estradiol or SERMs. Longer term trials may be possible with SERMs. Increasing the affinity of SERMs for estrogen receptors in the brain should increase the effectiveness of such strategies.
